# Prevalence and prognosis of acutely ill patients with organ failure at arrival to hospital: A systematic review

**DOI:** 10.1371/journal.pone.0206610

**Published:** 2018-11-01

**Authors:** Peter Bank Pedersen, Asbjørn Hrobjartsson, Daniel Lykke Nielsen, Daniel Pilsgaard Henriksen, Mikkel Brabrand, Annmarie Touborg Lassen

**Affiliations:** 1 Department of Emergency Medicine, Odense University Hospital, Odense, Denmark; 2 Institute of Clinical Research, University of Southern Denmark, Odense, Denmark; 3 Centre for Evidence-Based Medicine, University of Southern Denmark & Odense University Hospital, Odense, Denmark; 4 Department of Respiratory Medicine, Odense University Hospital, Odense, Denmark; 5 Department of Emergency Medicine, Hospital of South West Jutland, Esbjerg, Denmark; University of Mississippi Medical Center, UNITED STATES

## Abstract

**Introduction:**

Patients in an emergency department are diverse. Some are more seriously ill than others and some even arrive in multi-organ failure. Knowledge of the prevalence of organ failure and its prognosis in unselected patients is important from a diagnostic, hospital planning, and from a quality evaluation point of view, but is not reported systematically.

**Objectives:**

To analyse the prevalence and prognosis of new onset organ failure in unselected acute patients at arrival to hospital.

**Methods:**

A systematic review of studies of prevalence and prognosis of acutely ill patients with organ failure at arrival to hospital. We searched PubMed, Cochrane Library, Embase and Cinahl, and read references in included studies. Two authors decided independently on study eligibility and extracted data. Results were summarised qualitatively.

**Results:**

Four studies were included with a total of 678,960 patients. The number of different organ failures reported in the studies ranged from one to six, and the settings were emergency departments and wards. The definitions of organ failure varied between studies. The prevalence of organ failure was 7%, 14%, 14%, and 23%, and in-hospital mortality was 5%, 11% and 15% respectively. The relative risk of in-hospital mortality for patients with organ failure compared to patients without organ failure varied from 2.58 to 8.65. Numbers of organ failures per 1,000 visits varied from 71 to 256.

**Conclusion:**

The results of this review indicate that clinicians have good reasons to be alert when a patient arrives to the emergency department; as a state of organ failure seems both frequent and highly severe.

However, most studies identified were performed in patients after a diagnosis was established, and only very few studies were performed in unselected patients.

**Systematic review registration number:**

PROSPERO: CRD42017060871.

## Introduction

### Rationale

The unselected patients who attend an Emergency Department(ED) are a heterogeneous group, and identifying the critically ill as soon as possible is crucial. Some patients have one or more organ failures with diverse severity, aetiologies, and affecting different organs. Knowledge of the prevalence and prognosis for these patients are relevant to all healthcare workers in contact with critically ill patients, and are important from a diagnostic, hospital planning, and from a quality point of view, but is not reported systematically. Furthermore definitions of organ failure appear diverse based on different scores or systems [1–3]. Most studies with focus on organ failure are in selected groups of patients, but before patients arrive to the wards or Intensive Care Unit (ICU), most of them have passed through the ED, where the primary evaluation and treatment is performed.

Prevalence of organ failure has been demonstrated in 51–72% of patients in the ICU, mortality was 40–60% in one to five years, and early improvement in organ function improved prognosis [4–7]. Similar results are found in sepsis patients, where organ failure was a risk factor of deterioration [8, 9], and organ failure was persistent and long-term [10].

An efficient method of identifying patients with organ failure has yet to be discovered, but is very much needed, because patients with overt clinical evidence of organ dysfunction receive better care than patients without a clear presentation of their organ failure [11]. It is essential for these acutely ill patients, with organ failure, to be identified and treated as early as possible, preferably in the emergency department, or even in the prehospital setting to prevent deterioration, ICU-transfer, death or long-term morbidity. This has the potential to improve both short-term and long-term prognosis, in these patients [6, 9].

With this review we wanted to highlight knowledge in the early identification of organ failure patients at the hospital doorstep, with the aim to guide future optimisation of the organisation there meets these omnipresent patients.

### Objectives

Our main research objectives were to assess the prevalence and prognosis of new onset organ failures in acutely ill patients at arrival.

## Methods

Methods were described and published in our protocol developed ahead of this systematic review, and covered below [12].

### Protocol and registration

This systematic review is reported according to the Preferred Reporting Items for Systematic Reviews and Meta-Analyses: The PRISMA Statement [13, 14]. To describe the conduct, a protocol was developed in advance according to the Preferred Reporting Items for Systematic Reviews and Meta-Analysis Protocols (PRISMA-P) 2015 statement, and with inspiration from the Cochrane Handbook for Systematic Reviews of Interventions [15–17]. The protocol was registered in PROSPERO, as proposed in the reporting guideline, registration number: CRD42017060871.

### Eligibility criteria

#### Study designs

Eligible studies were randomized and non-randomized controlled trials and observational studies, assessing the prevalence of new organ failure, or prognosis of acutely ill patients, at arrival. Case reports and studies with less than 100 patients were excluded.

#### Participants/Population

We included studies on acutely ill adult patients (adult age limit set by included studies), with one or more of following organ failures: circulatory, respiratory, renal, cerebral, hepatic or coagulation failure. Studies on specific condition or disease were excluded, and studies on both children and adults were eligible, as long as adult data were presented.

#### Outcomes

We included studies on acutely ill adult patients (adult age limit set by included studies), with one or more of following organ failures: circulatory, respiratory, renal, cerebral, hepatic or coagulation failure. Studies on specific condition or disease were excluded, and studies on both children and adults were eligible, as long as adult data were presented.

#### Setting

We included studies of patients, who arrived at an emergency department, an acute medical unit, a trauma centre, a general ward, or other entrances for acutely ill patients. Studies were excluded if patients arrived directly at an intensive care unit.

#### Language

The search were performed without any language filtering, but the included words were all English, and publications in other languages were included in our search, but none were eligible.

### Information sources

Electronic databases, references in included studies and authors’ personal files, were searched. The databases were: PubMed, Cochrane Library, Embase and Cinahl, and the protocol database, PROSPERO, were searched for ongoing or recently completed systematic reviews on similar topics. The last search was run on 15 November 2017.

### Search

Search strategy was developed by input from authors, and with help form an information specialist from The Medical Research Library at University of Southern Denmark.

The reference lists of included studies were scanned for eligible studies. A similar search was performed at all databases, only adapted the exact subjects and syntax, which fit in the particular database. A research record table is included in S1 Table.

### Study selection

One author performed title and abstract screening, and excluded obviously ineligible studies. Remaining studies were read in full length, independent and in duplicate, by two review authors. Disagreements were discussed, at a face to face meeting. Reasons for excluding full text studies were documented and presented in S2 Table.

For evaluation of an included study, conducted in our own research unit, we contacted a researcher outside our unit to double-check the inclusion, to minimize the potential bias.

### Data collection process

We contacted the authors of the study conducted in our research unit and were provided with additional information and data. Data from other included studies were collected directly from the publication and some processing was performed to fill in missing data according to our table, and by transforming percentages into numbers and numbers into percentages.

### Data items

From included studies bibliographical and study description data, patient characteristics, and data related to prevalence and prognosis of organ failure, were extracted, using a data extraction form.

### Risk of bias in individual studies

To assess the risk of bias within included studies, the Quality in Prognosis Studies (QUIPS) tool for prognostic studies and the Newcastle-Ottawa Scale (NOS) for observational studies were used. The QUIPS tool rates 6 bias domains; study participation, study attrition, prognostic factor measurement, outcome measurement, study confounding and statistical analysis and reporting, as having high, moderate, or low risk of bias.[18] The NOS evaluate selection, comparability and outcome in case-control and cohort studies by assigning stars.[19] Two authors assessed every included study for bias independently, neither of the assessors was blinded to the studies, and disagreements were resolved by discussion. For studies describing both prevalence and prognosis, both risks of bias assessment tools were used.

### Planned methods of analysis

Reasons for heterogeneity according to clinical (participants and outcomes) and methodological (design and risk of bias) characteristics were explored. On the basis of scoping searches prior to protocol development, we did not expect to perform meta-analysis due to anticipated heterogeneity. However, we performed statistical test for heterogeneity (Cochran’s Q) and degree of heterogeneity were described with the I-square statistic, and because of substantial and statistically significant heterogeneity (I-square >60%, P<0.10), a meta-analysis with the aim of assessing a weighted estimate were not performed [20]. Statistical analyses were performed in Stata version 15.

A qualitative synthesis of the included studies based on tables and graphs, to sum up results and findings were presented [21].

In a subgroup of patients with circulatory failure, sub-group descriptions were performed based at available data.

### Confidence in cumulative evidence

Certainty of evidence was provided, inspired by the Grading of Recommendations Assessment (GRADE approach) depending on the basic design of the included studies and any downgrading decisions as: high, moderate, low or very low. Following assessment criteria were used, when considering downgrading the certainty of the evidence: risk of bias, inconsistency, indirectness, imprecision, and publication bias to decide how to grade the certainty of evidence [22, 23]. The assessments were performed by two authors, independently, and disagreements were resolved by discussion.

## Results

### Study selection

**Fig 1:**
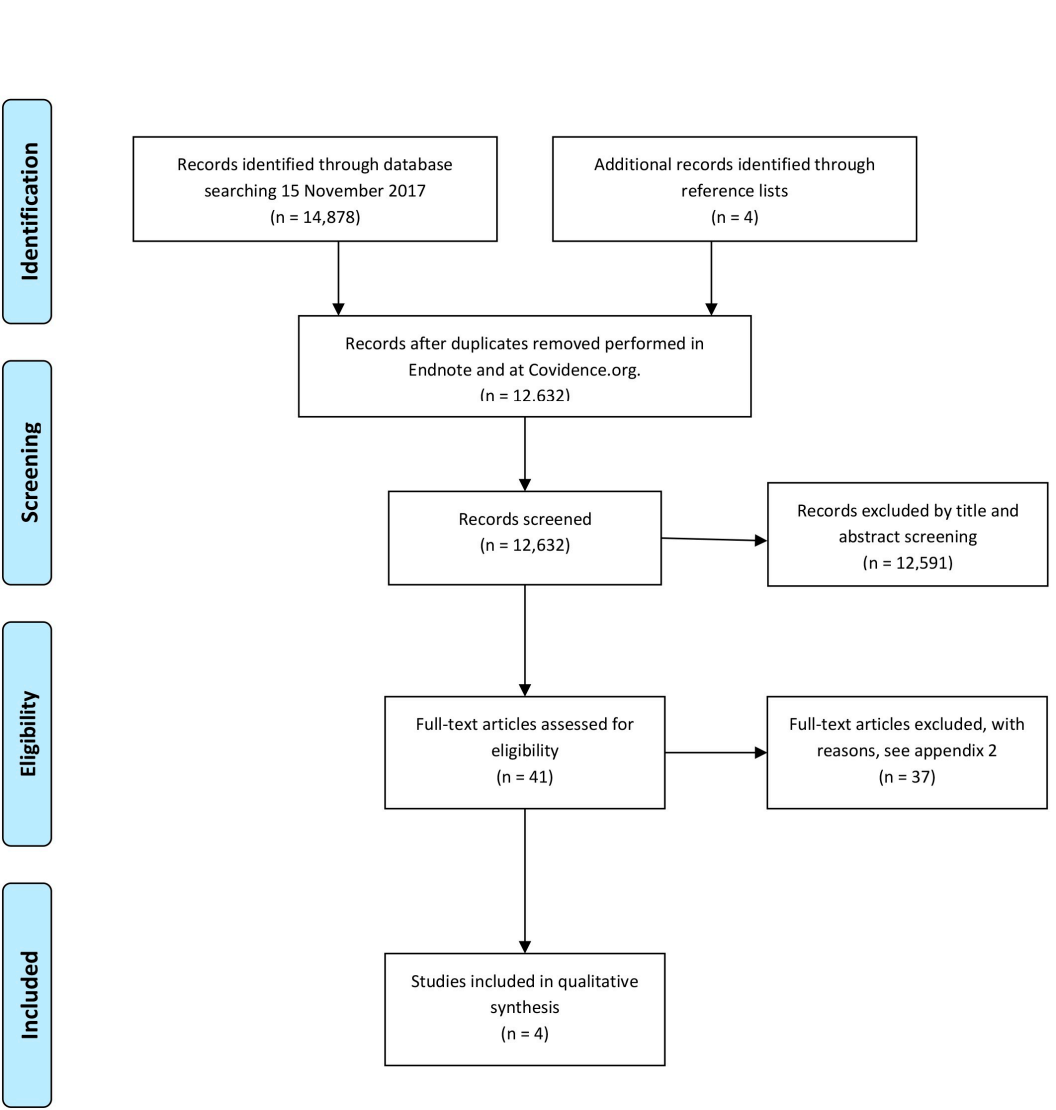
[28]: PRISMA-flow diagram of selection process, from search to inclusion.

The search conducted 15 November 2017 provided a total of 14,878 citations. Another four citations were identified through reference lists of included studies. Forty-one articles were read in full length and Appendix 2 provides an overview of full-text screening and reasons for excluding articles from the review. The proportion of agreement was 90.2% between the two Authors performing full-text screening. Thirty-seven studies did not meet the inclusion criteria, or fell under the exclusion criteria, leaving four studies for inclusion in the systematic review Fig 1 [24–27]. No unpublished relevant studies were obtained, and the PROSPERO search identified no reviews on similar topics.

### Study characteristics

The four included studies were cohort studies published in English between 2013 and 2015. Two studies were conducted in the United States and two in Europe, Denmark and United Kingdom, and the inclusion periods were between 14 days and four years.

The studies included covered a total population of 678,960 patients, and between 45.8% and 60% were males.

The numbers of organ failures included in the studies ranged from one to six. The prognostic outcome also varied; three studies presented in-hospital mortality and one study presented ICU-transfer; while the last study presented no prognostic outcome.

The settings were emergency departments and wards, and the different organ failure definitions for each study, are provided in S1 Text (Table 1) [24–27].

**Table 1:** Characteristics of the four included studies, for organ failure definitions, see S1 Text.

Study	Year	Country	Design	Period	Setting	Population, N	Age	Gender	Type of organ failure included
Benns et al	2013	United States of America	Cohort study	2006	Trauma and non-trauma centres in 38 US states	396,276 injured patients	64 (IQR 42–81), median	Male 48%	Respiratory, Circulatory, Hepatic, Renal
Challiner et al	2014	United Kingdom	Cohort study	2 x 7 days, September and February	Emergency admissions to Manchester Royal Infirmary	745 emergency admissions	NS	Male 54.6%	Renal
Churpek et al	2015	United States of America	Cohort study	November 2008—January 2013	Wards at five University Hospitals	269,951 ward patients	60/61, mean	Male 60%	Cerebral, Coagulatory, Renal, Respiratory, Circulatory, Hepatic
Lindvig et al	2014	Denmark	Cohort study	August 2009—August 2011	Acute Medical Ward at Odense University Hospital	11,988 first time admissions	66 (range 15–103), median	Male 45.8%	Renal, Respiratory, Circulatory

### Results of individual studies

The four studies included presented organ failure prevalence ranging from 6.5% to 23.1% (Table 2). Only three studies presented mortality data on organ failure patients, and none presented longer follow up than in-hospital. The in-hospital mortality ranged from 5.3% to 14.7% in the group of patients with organ failure and from 1.1% to 4.4% in the patients without organ failure (Table 2) [24–27].

**Table 2:** Results of individual studies, total population included, prevalence of organ failure, in-hospital mortality and relative risk of death for patients with compared to patient without organ failure. NS = Not specified.

Study	Year	Population, N	Population, patients with organ failure, total N (%)	In-hospital mortality, patients with organ failure, N (%)	In-hospital mortality, patients without organ failure, N (%)	Relative risk, organ failure/no organ failure, in-hospital mortality, (Cl 95%)
Benns et al	2013	396,276	25,758 (6.5)	3,788 (14.7)	6,299 (1.7)	8.65 (8.32–8.99)
Challiner et al	2014	745	106 (14.2)	12 (11.3)	28 (4.4)	2.58 (1.36–4.92)
Churpek et al	2015	269,951	36,767 (13.6)	1,934 (5.3)	2,565 (1.1)	4.78 (4.51–5.07)
Lindvig et al	2014	11,988	2,769 (23.1)	NS	NS	NS

### Syntheses of results

**Fig 2:**
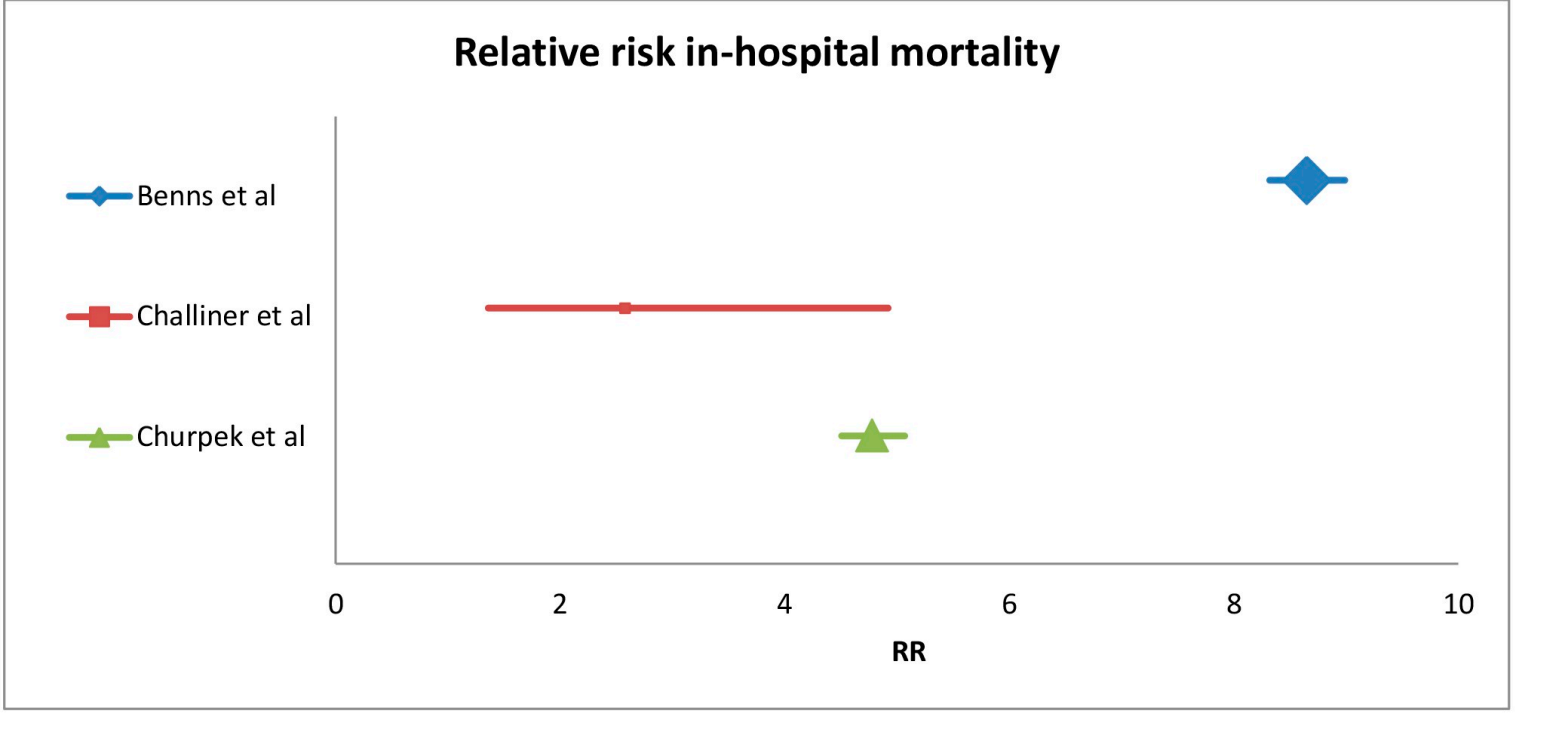
Forrest plot, relative risk of in-hospital mortality, patients with organ failure compared to patients without organ failure.

Significant heterogeneity between studies in both analyses was detected, prevalence; I^2^ = 100%, and mortality; I^2^ = 99.3%, and according to predefined criteria, no meta-analysis was performed. Relative risk of in-hospital mortality for patients with organ failure compared to patients without is presented in Table 2 and Fig 2.

Numbers of organ failures were calculated per 1,000 visits and varied from 71 to 256. Prevalence and mortality for patients with one to at least four organ failures are presented in Table 3, calculated based on data from included studies, and from prevalence-data collected from the authors of Lindvig et al.

**Table 3:** Prevalence of organ failures per 1000 visits, patients with 1, 2, 3, and 4 organ failures, and in-hospital mortality for patients with 1, 2, 3, and 4 organ failures. NS = Not specified.

Author	Number of organ failures, N/1000 visits	1 organ failure, % (N)	2 organ failures, % (N)	3 organ failures, % (N)	4 organ failures, % (N)	1 organ failure, in-hospital mortality, % (N)	2 organ failures, in-hospital mortality, % (N)	3 organ failures, in-hospital mortality, % (N)	4 organ failures, in-hospital mortality, % (N)
Benns et al	71	92.3% (23771)	6.6% (1702)	1 % (259)	0.1% (26)	12.4% (2948)	39.4% (671)	59.6% (169)	NS
Challiner et al	142	100% (106)	NS	NS	NS	11.3% (12)	NS	NS	NS
Churpek et al	159	85.5% (31448)	12.6% (4627)	1.7% (617)	0.2% (75)	4.1% (1289)	10.4% (481)	21.3% (131)	44.6% (33)
Lindvig et al	256	89.8% (1486)	9.4% (261)	0.8% (22)	NS	NS	NS	NS	NS

Three of the included studies described circulatory organ failure, two published data on in-hospital mortality at 12.3% and 41.5% respectively. Further data on patients with organ failure following trauma, bleeding, cardiac or sepsis were not published in the included studies.

### Risk of bias within studies

All four studies reported prevalence of organ failure and three of the studies reported in-hospital mortality. They were judged as having low to moderate risk of bias S3 Table.

### Confidence in cumulative evidence

The outcomes prevalence, based on all four studies, and mortality, based on three studies, was graded as having low certainty of evidence, mainly because of the inconsistency between studies. The outcome ICU-transfer was also graded as low certainty of evidence due to serious risk of bias and few events, based on one study S4 Table.

## Discussion

Four eligible studies were included in our systematic review, which only provided an indication of an answer to our research question. The studies covered in total 678,960 patients from EDs and wards, with a prevalence of organ failure between 6.5% and 23.1%, and in-hospital mortality between 5.3% and 14.7%. As expected and without regard for the differences between included studies, prognosis worsens by increasing number of organ failures per patient.

Specific conditions or diseases, or arrival at ICU were the most frequent reasons for exclusion at full-text screening, particular studies on sepsis patients, trauma patients or patients with suspected infection.

Due to significant heterogeneity between studies, meta-analysis was not performed, but results were presented in text and tables. Overall the included studies were judged as having moderate risk of bias and the certainty of evidence were graded as low.

Organ failure appeared frequent and was a very serious condition, and the anticipation is that all health care providers in an acute setting meet organ failure patients frequently. The health care personnel in emergency departments have to pass attention to baseline health, comorbidities, and clinical presentation to recognize patients with organ failure. But as revealed in this review, lack of research prevented us from the possibility of presenting an exact estimate for prevalence and prognosis; the included studies were too heterogeneous.

Most important this review highlighted large knowledge gaps surrounding the prevalence and prognosis of these omnipresent patients with organ failure. This had the potential to bring forward where to put the efforts at the hospital doorstep to recognize and treat organ failure patients as early as possible. The mortality associated with organ failure, the risk of ICU-transfer and even an accepted and consistent definition of organ failures in the Emergency Department remains topics for future research.

To explain heterogeneity we were looking at study characteristics, and organ failure definitions turned out very different. Two studies used definitions adapted from the 2001 Sepsis consensus definitions, one study used three different classifications of acute kidney injury, where organ failure at arrival were not possible to extract from presented data, and one study defined organ failures based on ICD-9 diagnostic codes, where patients without obvious organ failure might be missed. Previous studies have revealed that sepsis diagnosis based on symptoms and clinical findings at arrival, had higher prevalence than discharge diagnosis [29].

Other characteristics which differed across studies were numbers of organ failures included, and the setting. Two studies included patients from the emergency department, and two studies included ward patients, furthermore the organ failures studied ranged from one to six. One study presented a very broad definition of acute kidney injury and included no further organ failure, while another included ward patients with six different organ failures. One study presented a prevalence of organ failure at 23.1%, based on unselected patients at the acute medical ward. Studies including patients with suspected infection in the ED, presented a prevalence of organ failure at 48.7% [30], where mortality was 3–6% [31, 32], the latter in accordance with one included study, but only half of another. In patients with sepsis, the prevalence of organ failure has been described between 17 and 47%, and mortality between 5% and 11% [33, 34], a finding in accordance with the results of this review. If narrowing the inclusion of patients to severe sepsis the in-hospital mortality increases, as expected [35–37].

Finally there could be some differences between pre-hospital care organisations in the studies, according to criteria for admission to ICU, number of available ICU-beds, and more seriously ill patients might be admitted directly to the biggest centres. This information is not described in the included studies.

Early improvement better the prognosis in ICU patients, and as presented in this review, with increasing number of organ failures, prognosis deteriorates [4–7]. Maybe focus at organ failure patients at arrival could reduce number of flowing organ failures, lower the severity, diminish need of ICU-transfer, and thereby improve prognosis. Until further data are available, healthcare professionals in acute settings should bring careful attention and assessment to the presence of organ failure in the meeting with an acutely ill patient.

### Strengths

This systematic review was conducted on the basis of an accepted reporting guideline, and a protocol was created, registered and published in advance.

The electronic database search was very broad and was developed with help form an information specialist.

The full-text screening was performed independent and in duplicate, by two review authors, with a proportion of agreement at 90.2%. Likewise the data extraction, risk of bias assessment, and certainty of evidence grading were performed independent and in duplicate by two review authors.

### Limitations

Eligible studies indexed in searched electronic databases and references of included studies were discovered, but studies might be missed if indexed in other electronic databases.

Only one author conducted title and abstract screening, and when only four studies were included in final analysis, this revealed a risk of bias if just one or two eligible studies were missed. However, we did not identify any of such studies in the reference lists of the included papers.

Furthermore; we were not able to conduct a meta-analysis due to heterogeneity between studies, and we did not contact authors of three included studies to provide complete data, and data to complete planned sub-group analyses.

One study provided data on acute kidney injury instead of failure, and it was not possible to limit data extraction to failure.

## Conclusion

The results of this review indicate that clinicians have good reasons to be alert when a patient arrives to the emergency department; as a state of organ failure seems both frequent and highly severe.

However, most studies identified were performed in patients after a diagnosis was established, and only very few studies were performed in unselected patients.

## Supporting information

S1 Checklist (DOC)

S1 Text Organ failure definitions for each study included in the systematic review. (DOCX)

S1 Table Full search for Prevalence and Prognosis of Acutely Ill Patients with Organ Failure at Arrival: A Systematic Review. (DOCX)

S2 Table Full-text screening with reasons for exclusion. (DOCX)

S3 Table Risk of bias assessment. (DOCX)

S4 Table Quality of evidence, GRADE. (DOCX)
