# Supplementary material for: Prevalence and prognosis of acutely ill patients with organ failure at arrival to hospital: A systematic review
Source: PLoS One. 2018 Nov 1;13(11):e0206610. doi: 10.1371/journal.pone.0206610 (PMC6211733; doi:10.1371/journal.pone.0206610)
Supplement: S1 Text — (DOCX) [file pone.0206610.s002.docx]

**S1 Text: Organ failure definitions for each study included in the systematic review.**

| Benns et al: |
| --- |
| ICD-9 Diagnostic Codes to Define Organ Dysfunction System ICD-9 Code Description Renal  586 Renal failure, unspecified 584.9 Acute renal failure, unspecified 593.9 Unspecified disorder of kidney 585 Chronic renal failure (exclusion) 39.95 Hemodialysis Respiratory  518.4 Acute pulmonary edema 518.5 Pulmonary insufficiency following trauma 518.7 Transfusion related acute lung injury 518.81 Acute respiratory failure 518.82 Acute pulmonary insufficiency 518.84 Acute-on-chronic respiratory failure 518.83 Chronic respiratory failure (exclusion) 96.7 Mechanical ventilation Hepatic  570 Acute hepatic failure 782.4 Jaundice, unspecified 277.4 Hyperbilirubinemia 571 Chronic liver disease (exclusion) Cardiac  458 Hypotension 785.5 Nonhemorrhagic shock 998.0 Postoperative shock |

| Challiner et al | |  | | | |  | |  |  |  | |  |
| --- | --- | --- | --- | --- | --- | --- | --- | --- | --- | --- | --- | --- |
| RIFLE | | | | | AKIN | | | | | AKIB | | |
| Stage | Serum creatinine criteria | | MDRD eGFR criteria | Anuria criteria | Stage | | Aerum creatinine | | | Stage |  | |
| Risk | >1.5× baseline over 7 days | | >25% decrease over 7 days |  | 1 | | > 1.5× baseline or >26 mmol/l increase over 48 hours | | | 1 | >26 mmol/l increase over 24 h or >44 mmol/l over 48 h | |
| Injury | >2× baseline over 7 days | | >50% decrease over 7 days |  | 2 | | >2× baseline over 48 hours | | | 2 | >44 mmol/l increase over 24 h or >88 mmol/l over 48 h | |
| Failure | >3× baseline over 7 days | | >75% decrease over 7 days | anuria for >12 h | 3 | | >3× baseline or 44 mmol/l increase if baseline > 300 mmol/l over 48 hours or any RRT or anuria for >12 h | | | 3 | >88 mmol/l increase over 24 h or >132 mmol/l over 48 h | |

| Churpek et al |
| --- |
| Adapted from the 2001 Sepsis consensus definitions: Cerebral: (an observation of “responds to voice,” “responds to pain,” or “unresponsive” on the AVPU scale), Coagulatory (a platelet count <100 k/mm3), Renal (a creatinine value >2.0 mg/dl), Respiratory (a respiratory rate >24 breaths/min, or an oxygen saturation reading <90%), Circulatory (a systolic blood pressure reading <90 mm Hg), and Hepatic (a total bilirubin value >4 mg/dl) |

| Lindvig et al |
| --- |
| Renal = Creatinine ≥177 µmol/L, Respiratory = PaO2 ≤10.0 kPa, Circulatory = sBT ≤90 mmHg |
