# Supplementary material for: Prevalence and prognosis of acutely ill patients with organ failure at arrival to hospital: A systematic review
Source: PLoS One. 2018 Nov 1;13(11):e0206610. doi: 10.1371/journal.pone.0206610 (PMC6211733; doi:10.1371/journal.pone.0206610)
Supplement: S1 Table — (DOCX) [file pone.0206610.s003.docx]

**S1 Table: Full search for Prevalence and Prognosis of Acutely Ill Patients with Organ Failure at Arrival: A Systematic Review**

| Date 15/11-2017 | Search: PubMed | Hits |
| --- | --- | --- |
| #1 | Multiple Organ Failure[Mesh] | 9814 |
| #2 | Respiratory Insufficiency[Mesh] | 57761 |
| #3 | Renal Insufficiency[Mesh] | 149538 |
| #4 | Cognitive Dysfunction[Mesh] | 6299 |
| #5 | Shock[Mesh] | 68410 |
| #6 | Liver Failure[Mesh] | 22123 |
| #7 | Hepatic Insufficiency[Mesh] | 22704 |
| #8 | ("Organ failure" OR "Organ failures" OR "Organ dysfunction" OR "Organ dysfunctions" OR "Organ system dysfunction" OR "Organ system dysfunctions" OR "Multiple organ failure" OR "Respiratory insufficiency" OR "Respiratory failure" OR "Acute respiratory failure" OR "Respiratory insufficiencies" OR "Respiratory failures" OR "Renal insufficiency" OR "Renal insufficiencies" OR "Kidney insufficiency" OR "Kidney insufficiencies" OR "Kidney failure" OR "Acute kidney failure" OR "Renal failure" OR "Renal failures" OR "Cognitive dysfunction" OR "Cognitive dysfunctions" OR "Mental deterioration" OR "Mental deteriorations" OR "Brain dysfunction" OR "Shock" OR "Circulatory failure" OR "Circulatory collapse" OR "Liver failure" OR "Acute liver failure" OR "Hepatic failure" OR "Hepatic insufficiency" OR "Liver insufficiency" OR "Coagulopathy" OR "Coagulation failure") | 523625 |
| #9 | #1 OR #2 OR #3 OR #4 OR #5 OR #6 OR #7 OR #8 | 577586 |
| #10 | ("Emergency department" OR "Emergency departments" OR "Emergency unit" OR "Emergency units" OR "Emergency ward" OR "Emergency wards" OR "Emergency room" OR "Emergency rooms" OR "Acute medical unit" OR "Acute medical units" OR "Non-ICU") | 94869 |
| #11 | #9 AND #10 | 5921 |

| Date 15/11-2017 | Search: Embase | Hits |
| --- | --- | --- |
| #1 | Multiple organ failure | 42191 |
| #2 | Respiratory failure | 94818 |
| #3 | Acute respiratory failure | 9983 |
| #4 | Kidney failure | 147736 |
| #5 | Acute kidney failure | 111458 |
| #6 | Brain dysfunction | 37507 |
| #7 | Shock | 55743 |
| #8 | Liver failure | 45153 |
| #9 | Acute liver failure | 10986 |
| #10 | Organ failure/ OR Organ failures/ OR Organ dysfunction/ OR Organ dysfunctions/ OR Organ system dysfunction/ OR Organ system dysfunctions/ OR Multiple organ failure/ OR Respiratory insufficiency/ OR Respiratory failure/ OR Acute respiratory failure/ OR Respiratory insufficiencies/ OR Respiratory failures/ OR Renal insufficiency/ OR Renal insufficiencies/ OR Kidney insufficiency/ OR Kidney insufficiencies/ OR Kidney failure/ OR Acute kidney failure/ OR Renal failure/ OR Renal failures/ OR Cognitive dysfunction/ OR Cognitive dysfunctions/ OR Mental deterioration/ OR Mental deteriorations/ OR Brain dysfunction/ OR Shock/ OR Circulatory failure/ OR Circulatory collapse/ OR Liver failure/ OR Acute liver failure/ OR Hepatic failure/ OR Hepatic insufficiency/ OR Liver insufficiency/ OR Coagulopathy/ OR Coagulation failure/ | 787427 |
| #11 | #1 OR #2 OR #3 OR #4 OR #5 OR #6 OR #7 OR #8 OR #9 OR #10 | 787427 |
| #12 | Emergency ward | 165297 |
| #13 | Emergency department/ OR Emergency departments/ OR Emergency unit/ OR Emergency units/ OR Emergency ward/ OR Emergency wards/ OR Emergency room/ OR Emergency rooms/ OR Acute medical unit/ OR Acute medical units/ OR Non-ICU/ | 165306 |
| #14 | #12 OR #13 | 165306 |
| #15 | #11 AND #14 | 6985 |

| Date 15/11-2017 | Search: Cinahl | Hits |
| --- | --- | --- |
| #1 | Respiratory failure | 7552 |
| #2 | Renal insufficiency | 18548 |
| #3 | Shock | 6591 |
| #4 | Liver failure | 1468 |
| #5 | Organ failure OR Organ failures OR Organ dysfunction OR Organ dysfunctions OR Organ system dysfunction OR Organ system dysfunctions OR Multiple organ failure OR Respiratory insufficiency OR Respiratory failure OR Acute respiratory failure OR Respiratory insufficiencies OR Respiratory failures OR Renal insufficiency OR Renal insufficiencies OR Kidney insufficiency OR Kidney insufficiencies OR Kidney failure OR Acute kidney failure OR Renal failure OR Renal failures OR Cognitive dysfunction OR Cognitive dysfunctions OR Mental deterioration OR Mental deteriorations OR Brain dysfunction OR Shock OR Circulatory failure OR Circulatory collapse OR Liver failure OR Acute liver failure OR Hepatic failure OR Hepatic insufficiency OR Liver insufficiency OR Coagulopathy OR Coagulation failure | 47644 |
| #6 | #1 OR #2 OR #3 OR #4 OR #5 | 52416 |
| #7 | Emergency department OR Emergency departments OR Emergency unit OR Emergency units OR Emergency ward OR Emergency wards OR Emergency room OR Emergency rooms OR Acute medical unit OR Acute medical units OR Non-ICU | 32550 |
| #8 | #6 AND #7 | 1173 |

| Date 15/11-2017 | Search: Cochrane Library | Hits |
| --- | --- | --- |
| #1 | Multiple Organ Failure[Mesh] | 369 |
| #2 | Respiratory Insufficiency[Mesh] | 2035 |
| #3 | Renal Insufficiency[Mesh] | 6232 |
| #4 | Shock[Mesh] | 1615 |
| #5 | Liver Failure[Mesh] | 646 |
| #6 | Hepatic Insufficiency[Mesh] | 675 |
| #7 | ("Organ failure" OR "Organ failures" OR "Organ dysfunction" OR "Organ dysfunctions" OR "Organ system dysfunction" OR "Organ system dysfunctions" OR "Multiple organ failure" OR "Respiratory insufficiency" OR "Respiratory failure" OR "Acute respiratory failure" OR "Respiratory insufficiencies" OR "Respiratory failures" OR "Renal insufficiency" OR "Renal insufficiencies" OR "Kidney insufficiency" OR "Kidney insufficiencies" OR "Kidney failure" OR "Acute kidney failure" OR "Renal failure" OR "Renal failures" OR "Cognitive dysfunction" OR "Cognitive dysfunctions" OR "Mental deterioration" OR "Mental deteriorations" OR "Brain dysfunction" OR "Shock" OR "Circulatory failure" OR "Circulatory collapse" OR "Liver failure" OR "Acute liver failure" OR "Hepatic failure" OR "Hepatic insufficiency" OR "Liver insufficiency" OR "Coagulopathy" OR "Coagulation failure") | 30204 |
| #8 | #1 OR #2 OR #3 OR #4 OR #5 OR #6 OR #7 | 32202 |
| #9 | ("Emergency department" OR "Emergency departments" OR "Emergency unit" OR "Emergency units" OR "Emergency ward" OR "Emergency wards" OR "Emergency room" OR "Emergency rooms" OR "Acute medical unit" OR "Acute medical units" OR "Non-ICU") | 9562 |
| #10 | #8 AND #9 | 799 |
