# Supplementary material for: Prevalence and prognosis of acutely ill patients with organ failure at arrival to hospital: A systematic review
Source: PLoS One. 2018 Nov 1;13(11):e0206610. doi: 10.1371/journal.pone.0206610 (PMC6211733; doi:10.1371/journal.pone.0206610)
Supplement: S2 Table — (DOCX) [file pone.0206610.s004.docx]

S2 Table: Full-text screening with reasons for exclusion.

|  |  |  |  |  | **Inclusion criteria** |  |  |  |  | **Exclusion criteria** |  |  |  |  |
| --- | --- | --- | --- | --- | --- | --- | --- | --- | --- | --- | --- | --- | --- | --- |
| **Title** | **Year** | **Country** | **Inclusion** | **Reason** | Acutely ill adult patients | Organ failure | Prevalence or ICU-transfer or mortality | Arrival non-ICU | Readable language | Arrival ICU | < 100 patients | Case Report | Only data on patients with specific condition or disease | No separate adult data |
| Evaluating the efficacy of a risk predictor panel in identifying patients at elevated risk of morbidity following emergency admission | 2016 | United Kingdom | No | Adult patients? No data on organ failure | ? | % | X | X | X | X | X | X | X | ? |
| Sepsis clinical criteria in Emergency Department patients admitted to an Intensive Care Unit: An external validation study of Quick Sequential Organ Failure Assessment | 2016 | United States of America | No | Arrival ICU Only data on patients with Sepsis | X | X | X | % | X | X | X | X | % | X |
| Heparin-binding Protein Is A Predictor Of Progressive Organ Dysfunction In Emergency Department Sepsis Patients | 2014 | United States of America & Sweden | No | Only data on patients with suspected infection | X | X | X | X | X | X | X | X | % | X |
| Epidemiology of Trauma Deaths | 1980 | United States of America | No | Not at arrival Only data on trauma patients | X | X | X | % | X | X | X | X | % | X |
| Benchmarking the incidence of organ failure after injury at trauma centres and nontrauma centres in the United States | 2013 | United States of America | Yes |  | X | X | X | X | X | X | X | X | X | X |
| Shock index as a predictor of ICU-transfer in patients admitted to the medical ward with sepsis | 2015 | ? | No | Only data on patients with sepsis | ? | X | X | X | X | X | X | X | % | ? |
| Detection and clinical management of the systemic inflammatory response syndrome in medicine emergency room. 24 hours follow-up in a general hospital | 2008 | Spain | No | Only data on patients with SIRS Organ failure unknown Only in Spanish | ? | ? | X | X | X | X | X | X | % | ? |
| Utility of the Shock Index in Predicting Mortality in Traumatically Injured Patients | 2009 | United States of America | No | No data on organ failure No separate adult data Only data on patients with trauma | % | % | X | X | X | X | X | X | % | % |
| Incidence and consequence of acute kidney injury in unselected emergency admissions to a large acute UK hospital trust | 2014 | United Kingdom | Yes |  | X | X | X | X | X | X | X | X | X | X |
| Risk stratification and prognostic performance of the predisposition, infection, response, and organ dysfunction (PIRO) scoring system in septic patients in the emergency department: a cohort study | 2014 | China | No | Only data on patients with sepsis | X | X | X | X | X | X | X | X | % | X |
| Quick Sepsis-related Organ Failure Assessment, Systemic Inflammatory Response Syndrome, and Early Warning Scores for Detecting Clinical Deterioration in Infected Patients outside the Intensive Care Unit | 2017 | United States of America | No | No data on organ failure Only data on patients with suspected infection | X | % | X | X | X | X | X | X | % | X |
| Incidence and Prognostic Value of the Systemic Inflammatory Response Syndrome and Organ Dysfunctions in Ward Patients | 2015 | United States of America | Yes |  | X | X | X | X | X | X | X | X | X | X |
| Critical Role of Activated Protein C in Early Coagulopathy and Later Organ Failure, Infection and Death in Trauma Patients | 2012 | United States of America | No | Only data on patients with major trauma | X | X | X | X | X | X | X | X | % | X |
| The prognostic performance of the predisposition, infection, response and organ failure (PIRO) classification in high-risk and low-risk emergency department sepsis populations: comparison with clinical judgement and sepsis category | 2017 | The Netherlands | No | Only data on patients with sepsis or suspected infection | X | X | X | X | X | X | X | X | % | X |
| Severe Sepsis in the Emergency Department - An Observational Cohort Study from the University Hospital of the West Indies | 2013 | Jamaica | No | No data on organ failure, only data on patients with sepsis | X | % | X | X | X | X | X | X | % | X |
| Comparison of qSOFA and SIRS for predicting adverse outcomes of patients with suspicion of sepsis outside the intensive care unit | 2017 | United States of America | No | Only data on patients with sepsis and admission to ICU | X | X | X | % | X | % | X | X | % | X |
| Assessment of Clinical Deterioration and Progressive Organ Failure in Moderate Severity Emergency Department Sepsis Patients | 2012 | United States of America | No | Only data on sepsis patients in PRE-SHOCK condition | X | X | X | X | X | X | X | X | % | X |
| The long-term burden of severe sepsis and septic shock: Sepsis recidivism and organ dysfunction | 2016 | United States of America | No | Only data on patients with severe sepsis or septic shock | X | X | X | X | X | X | X | X | % | X |
| Time to initial antibiotic administration, and short-term mortality among patients admitted with community-acquired severe infections with and without the presence of systemic inflammatory response syndrome: a follow-up study | 2015 | Denmark | No | Only data on patients with severe infection | X | X | X | X | X | X | X | X | % | X |
| Incidence Rate of Community-Acquired Sepsis Among Hospitalized Acute Medical Patients-A Population-Based Survey | 2014 | Denmark | No | Only data on patients with sepsis Patients arriving ICU included | X | X | X | % | X | % | X | X | % | X |
| Predictors of early progression to severe sepsis or shock among emergency department patients with nonsevere sepsis | 2016 | United States of America | No | Only data on patients with non-severe sepsis at arrival | ? | X | X | X | X | X | X | X | % | X |
| Etiology of Shock in the Emergency Department; A 12 year population based cohort study | 2016 | Denmark | No | Only data on patients with shock | X | X | X | X | X | X | X | X | % | X |
| Proof of principle: The predisposition, infection, response, organ failure sepsis staging system | 2011 | United States of America | No | Only data on patients with suspected infection | X | X | X | X | X | X | X | X | % | X |
| Number of organ dysfunctions predicts mortality in emergency department patients with suspected infection: a multicentre validation study | 2015 | United States of America & Denmark | No | Only data on patients with suspected infection The external validation patients are "selected" | X | X | X | X | X | X | X | X | % | X |
| Increasing number of organ dysfunctions is an excellent predictor of in-hospital mortality in emergency department patients with suspected infection: an internal and external prospective validation study | 2013 | United States of America & Denmark | No | Only data on patients with suspected infection | X | X | X | X | X | X | X | X | % | X |
| Lack of clinically evident signs of organ failure affects ED treatment of patients with severe sepsis | 2013 | The Netherlands | No | Only data on patients with severe sepsis or septic shock | X | X | X | X | X | X | X | X | % | X |
| Outcomes of the Surviving Sepsis Campaign in intensive care units in the USA and Europe: a prospective cohort study | 2012 | United States of America & Europe | No | Only data on patients with severe sepsis or septic shock No separate data on adults | ? | X | X | % | X | % | X | X | % | ? |
| Sensivity of Systemic Inflammatory Response Syndrome for Critical Illness Among Emergency Department Patients | 2014 | United States of America | No | No data on organ failure | X | % | X | X | X | X | X | X | X | X |
| How do bacteraemic patients present to the emergency department and what is the diagnostic validity of the clinical parameters; temperature, C-reactive protein and systemic inflammatory response syndrome? | 2014 | Denmark | Yes |  | X | X | X | X | X | X | X | X | X | X |
| Mortality and prognostic factors of patients who have blood cultures performed in the emergency department: a cohort study | 2016 | Denmark | No | Only data on blood-cultured patients | X | X | X | X | X | X | X | X | % | X |
| Comparison of PIRO, SOFA, and MEDS Scores for Predicting Mortality in Emergency Department Patients With Severe Sepsis and Septic Shock | 2014 | Australia | No | Only data on patients with sepsis | X | X | X | X | X | X | X | X | % | X |
| Serum Lactate Predicts Adverse Outcomes in Emergency Department Patients With and Without Infection | 2017 | United States of America | No | Only data on patients with lactate measurements and abnormal vital signs Minimum data on organ failure | X | ? | X | X | X | X | X | X | % | X |
| Sepsis patients in the emergency department: stratification using the Clinical Impression Score, Predisposition, Infection, Response and Organ dysfunction score or quick Sequential Organ Failure Assessment score? | 2017 | The Netherlands | No | Only data on patients with sepsis | X | X | X | X | X | X | X | X | % | X |
| Epidemiology of severe sepsis in the emergency department and difficulties in the initial assistance | 2008 | Brazil | No | Only data on patients with severe sepsis | X | X | X | X | X | X | X | X | % | X |
| The Epidemiology of Acute Organ System Dysfunction From Severe Sepsis Outside of the Intensive Care Unit | 2013 | United States of America | No | Only data on patients with severe sepsis | X | X | X | X | X | X | X | X | % | X |
| Denver Trauma Organ Failure Score Outperforms Traditional Methods of Risk Stratification in Trauma | 2012 | United States of America | No | Only data on trauma patients | X | X | X | X | X | X | X | X | % | X |
| The Association of Sepsis Syndrome and Organ Dysfunction With Mortality in Emergency Department Patients With Suspected Infection | 2006 | United States of America | No | Only data on blood-cultured patients | X | X | X | X | X | X | X | X | % | X |
| Quick SOFA Scores Predict Mortality in Adult Emergency Department Patients With and Without Suspected Infection | 2017 | United States of America | No | No data on organ failure | X | % | X | X | X | X | X | X | X | X |
| Prediction of postinjury multiple-organ failure in the emergency department: Development of the Denver Emergency Department Trauma Organ Failure Score | 2014 | United States of America | No | Only data on trauma patients | X | X | X | X | X | X | X | X | % | X |
| Validation of the Denver Emergency Department Trauma Organ Failure Score to Predict Post-Injury Multiple Organ Failure | 2016 | United States of America | No | Only data on trauma patients | X | X | X | X | X | X | X | X | % | X |
| Relationship between procalcitonin plasma levels and severity of injury, sepsis, organ failure , and mortality in injured patients | 2000 | Switzerland | No | Only patients with mechanical trauma and in need of surveillance in the ICU | X | X | X | ? | X | ? | X | X | % | X |
