# Supplementary material for: Prevalence and prognosis of acutely ill patients with organ failure at arrival to hospital: A systematic review
Source: PLoS One. 2018 Nov 1;13(11):e0206610. doi: 10.1371/journal.pone.0206610 (PMC6211733; doi:10.1371/journal.pone.0206610)
Supplement: S3 Table — (DOCX) [file pone.0206610.s005.docx]

**S3 Table: Risk of bias assessment.**

Risk of bias assessment regarding prevalence, using the Newcastle-Ottawa Scale by assessing stars, all studies judged as having moderate risk of bias.

| Newcastle-Ottawa Scale | |  |  |  |
| --- | --- | --- | --- | --- |
| Study | Selection | Comparability | Outcome | Total |
| Benns et al | * * * |  | * * | 5 (55.6%) |
| Challiner et al | * * * | * | * * * | 7 (77.8%) |
| Churpek et al | * * * |  | * * * | 6 (66.7%) |
| Lindvig et al | * * * |  | * * * | 6 (66.7%) |

Risk of bias assessment regarding prognostic factors, using the Quality in Prognosis Studies tool, studies judged as having low to moderate risk of bias.

| Quality in Prognosis Studies |  |  |  |  |  |  |
| --- | --- | --- | --- | --- | --- | --- |
| Study | Study Participation | Study Attrition | Prognostic Factor Measurement | Outcome Measurement | Study Confounding | Statistical Analysis and Reporting |
| Benns et al | Moderate | NA | Moderate | Low | Moderate | Low |
| Challiner et al | Moderate | NA | Low | Low | Moderate | Low |
| Churpek et al | Low | NA | Moderate | Low | Moderate | Low |
