# Supplementary material for: Prevalence and prognosis of acutely ill patients with organ failure at arrival to hospital: A systematic review
Source: PLoS One. 2018 Nov 1;13(11):e0206610. doi: 10.1371/journal.pone.0206610 (PMC6211733; doi:10.1371/journal.pone.0206610)
Supplement: S4 Table — (DOCX) [file pone.0206610.s006.docx]

**S4 Table: Quality of evidence, GRADE.**

Moderate quality of evidence at baseline, due to the fact that, the studies are observational cohort studies, for each outcome downgraded once; result is low quality of evidence.

| Outcome: ICU-transfer | Rating | Notes | Quality of the evidence |
| --- | --- | --- | --- |
| Risk of Bias | Serious | Serious risk of bias |  |
| Inconsistency | No | Only one study included |  |
| Indirectness | No | Not at all | ++oo |
| Imprecision | Serious | Few events | Low |
| Publication Bias | No | Undetected |  |
|  |  |  |  |
| Outcome: Mortality | Rating | Notes | Quality of the evidence |
| Risk of Bias | No | No serious risk of bias |  |
| Inconsistency | Very serious | Methodological heterogeneity |  |
| Indirectness | No | Not at all | ++oo |
| Imprecision | No | Not at all | Low |
| Publication Bias | No | Undetected |  |
|  |  |  |  |
| Outcome: Prevalence | Rating | Notes | Quality of the evidence |
| Risk of Bias | No | No serious risk of bias |  |
| Inconsistency | Very serious | Methodological heterogeneity |  |
| Indirectness | No | Not at all | ++oo |
| Imprecision | No | Not at all | Low |
| Publication Bias | No | Undetected |  |
